# Creative Commons licenses and the non-commercial condition: Implications for the re-use of biodiversity information

**DOI:** 10.3897/zookeys.150.2189

**Published:** 2011-11-28

**Authors:** Gregor Hagedorn, Daniel Mietchen, Robert A. Morris, Donat Agosti, Lyubomir Penev, Walter G. Berendsohn, Donald Hobern

**Affiliations:** 1 Julius Kühn-Institute, Federal Research Centre for Cultivated Plants, Königin-Luise-Str. 19, 14195 Berlin, Germany; 2 EvoMRI Communications, Zwätzengasse 10, 07743 Jena, Germany; Open Knowledge Foundation Deutschland, Prenzlauer Allee 217, 10405 Berlin; Germany, and Pensoft Publishers, 13a Geo Milev Str., Sofia, Bulgaria; 3 Harvard University Herbaria and University of Massachusetts at Boston; 4 Plazi, Zinggstr. 16, 3007 Bern, Switzerland; 5 Bulgarian Academy of Sciences & Pensoft Publishers, Sofia, Bulgaria; 6 Botanischer Garten und Botanisches Museum, Freie Universität Berlin, Königin-Luise-Straße 6-8, 14195 Berlin, Germany; 7 Atlas of Living Australia, CSIRO Ecosystem Sciences, GPO Box 1700, Canberra, ACT 2601, Australia

**Keywords:** Creative Commons, Open Access, Open Content, Licensing, Non-profit, Open Educational Resources, Data Sharing, Software Licenses, Europeana

## Abstract

The Creative Commons (CC) licenses are a suite of copyright-based licenses defining terms for the distribution and re-use of creative works. CC provides licenses for different use cases and includes open content licenses such as the Attribution license (CC BY, used by many Open Access scientific publishers) and the Attribution Share Alike license (CC BY-SA, used by Wikipedia, for example). However, the license suite also contains non-free and non-open licenses like those containing a “non-commercial” (NC) condition. Although many people identify “non-commercial” with “non-profit”, detailed analysis reveals that significant differences exist and that the license may impose some unexpected re-use limitations on works thus licensed. After providing background information on the concepts of Creative Commons licenses in general, this contribution focuses on the NC condition, its advantages, disadvantages and appropriate scope. Specifically, it contributes material towards a risk analysis for potential re-users of NC-licensed works.

## Copyright, Science, and Education

Copyright is a state-guaranteed right given to creators of “literary and artistic works” (see Art. 2 (1) of the Berne Convention, World Intellectual Property Organisation 1979) to control the reproduction, distribution, adaptation or translation of their works. The term “work” includes a wide array of forms of intellectual creations, including text, photographs, diagrams, maps, movies, etc. To be eligible for copyright, a work must be original, individual, singular and new (see, e.g., Agosti and Egloff 2009).

Copyright typically lasts 50 to 70 years after the death of the last contributor of a work. Under the standard term in the European Union (“life plus 70 years”) even very old works may require individual negotiations with the rights owners before they can be made accessible digitally or parts of them re-used in a new context. If contractual arrangements between contributors of a work and the death year of at least one potential rights owner are unknown, only works before perhaps 1871 can reasonably be assumed to be in the public domain.

The well-known Biodiversity Heritage Library (BHL, biodiversitylibrary.org), providing access to the published knowledge about the species of world, has somewhat more favorable conditions. Operating under U.S.A. legislation, most works published before 1923 as well as a significant number of works published between 1923 and 1978 (see Hirtle 2011) are no longer under copyright control. This makes the public domain in the U.S.A. exceptionally rich. After 1978, however, copyright duration has largely been increased to life plus 70 years. A continuing exception is, e.g., that works created by an officer or employee of the United States Government – including, e.g., the Food and Drug Administration and the National Institutes of Health – as part of that person’s official duties are in the public domain, irrespective of publication year. However, the success of BHL cannot be simply projected into the future.

For singular cultural works such as poems, novels, paintings, or musical compositions, and where a reproduction concerns major parts of the work, the balance offered by copyright law between the rights of creators and the rights of the public for creativity and innovation is widely considered reasonable. However, the balance may already be questionable when it comes to the creative or even unavoidable (background music or company logos visible in documentary movies) inclusions of fragments of copyrighted works. Increasing IPR management and risk avoidance by companies may create a stifling and suppressive environment (Aoki et al. 2006). In some jurisdictions exceptions for educational activities (Nabhan 2009; Seng 2009; Xalabarder 2009) or fair-use doctrines allow a limited re-use of small excerpts of copyrighted materials (see, e.g., the English Wikipedia). In many other jurisdictions this is, however, not permitted (see, e.g., the German or Japanese Wikipedias), severely limiting non-commercial efforts to provide educational materials.

Ideas, knowledge, inventions, information, or data are intentionally not copyright-protected (see, e.g., World Intellectual Property Organisation 1979). The public interest, e.g., to talk about "e = mc²" prior to 2025 (i.e. 70 years after the death of Einstein) is considered to outweigh the interest of scientists to be rewarded for their work. For the most parts, scientists have traditionally been satisfied with the moral right to be cited for their original work. In addition, technical inventions may also be protected by patents (with a much briefer protection period of mostly 20 years and explicit provisions for knowledge dissemination).

Unfortunately, in science and education, knowledge and data are often intermingled with copyrighted expressions of the same. Many publishers establish barriers to knowledge sharing by asserting copyright on non-copyrightable plain or formal expressions of that knowledge. In the area of biodiversity, often dealing with textually expressed data or data expressed in images, this is a major obstacle. Attempts are under way to establish special procedures to extract and disseminate the – non-copyrightable – knowledge that is included within protected works. One such procedure for textual expressions, operating under principles of Swiss law, is described in Agosti and Egloff (2009). Even more difficult is the situation for drawings, photographs or diagrams. Documentary photographs or drawings are widely granted full copyright status even if they depict entirely factual information that might not itself be copyrightable. In the case of biodiversity knowledge, where much data and knowledge is expressed in these documentary forms, this can be a substantial barrier to knowledge dissemination. Finally, copyright on diagrams may severely diminish the ability of teachers and educators to disseminate knowledge efficiently. For example, the results of publicly funded research on climate change may be published in a scientific journal that does not allow educators or teachers to re-use graphs or other materials for their teaching purposes.

Such problems could best be reduced by implementing appropriate restrictions to copyright protection or by establishing legal licenses for the use of works that serve primarily as expressions of knowledge (including diagrams or documentary photography) for educational or research purposes. Unfortunately, such restrictions can only be introduced by legislative acts and depend therefore on political bargaining.

## Open

Fortunately, many individuals and organizations in the scientific field, while legally entitled to complete and century-long copyright control, see advantages in less restrictive terms-of-use. In contrast to cultural works, the primary intent of scientific works is most often the dissemination of knowledge. Increasing this dissemination may thus be a principal goal. Alternatively, it may be seen a secondary goal because it improves the researchers’ reputation and the brand recognition of their institutions or research areas – factors that influence future chances of obtaining research funds.

Furthermore, society often funds research to foster innovation and the general welfare, or to address problems of critical societal interest such as climate change. The vast majority of costs for such research are paid up-front by research grants or institutional funds. Trying to generate relatively minute additional income by preventing public access to the results of publicly funded research is considered inappropriate by many.

One approach to increasing dissemination and public access is found in the Open Access (OA) movement, which aims to provide readers with free and unrestricted access to the scientific literature (Suber 2004-2010). Publications freely available to the reader are more frequently cited than publications behind a pay wall (Swan 2010). Furthermore, a growing number of funding agencies (see, e.g., Wellcome Trust 2003-2011, or the OA pilot of the Seventh Research Framework Programme, European Commission 2011), as well as the public, are asking for free and open access to the results of publicly-funded research.

The original Bethesda Open Access declaration (Brown et al. 2003) includes the right to create and distribute derivative works. Some OA journals, however, do not grant such rights. Parts of the research community, e.g. some authors choosing an OA journal, consider read-access sufficient. Based on anecdotal evidence, this may in part be because they erroneously believe that permissions for the most typical re-use scenarios (e.g., disseminating lecture materials that include other researcher’s published graphs or images on a conference website) are already granted. The majority of Open Access journals today, however, provide licenses that do indeed confer broad re-use rights. This gives the journals significant advantages in dissemination and broadens the researchers’ legal re-use options. It greatly simplifies the ways in which future works may build on existing ones (see, e.g., Bourne et al. 2008; MacCallum 2007).

Closely related to the Open Access movement is the Open Educational Resources movement (OER, see, e.g., Wilson-Strydom 2009; Butcher 2011). Clearly, re-use and adaptation rights are vital for educational resources (Keller and Mossink 2008).

Individuals or organizations desiring to grant Open Access and defined community re-use of their creative works can do so by creating their own individual terms-of-use. For the copyright owner and license giver (licensor), this may often appear to be the simplest and safest way to proceed. For re-users (licensees), however, legal advice may be required to assess whether individual terms-of-use permit their intended use in their own jurisdiction. Not doing so carries the risk of unwitting copyright violations that may result in legal action. The administrative and legal cost of handling a large diversity of such terms-of-use can become an impediment to re-use. At the same time, there is a substantial risk to the copyright holder that self-created terms-of-use licenses may have undesired outcomes, particularly when interpreted under multiple jurisdictions on a global scale.

Creative Commons (abbreviated CC) licenses have been created to address these problems. These licenses provide standardized terms-of-use definitions. Amongst the ca. 2.2 million articles deposited in PubMed Central, already about 10% are CC-licensed (U.S. National Library of Medicine 2011b; Björk et al. 2010).

## Creative Commons

Creative Commons is a US-based non-profit organization that authors, reviews, and publishes a suite of licenses defining standard options for the distribution and re-use of creative, copyrightable works. Together with its world-wide affiliate organizations and partners, it provides standardized and scrutinized license texts, translation into languages and adaptations for various jurisdictions (presently over 50 adaptations are provided). Creative Commons is never a party in the contract of such a license (other than in connection with materials for which it holds copyright itself).

**Figure:**
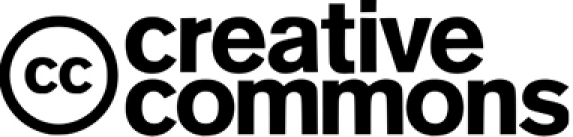
Creative Commons Logo, CC BY, Creative Commons

The roots of the organization go back several years before the actual foundation. In 1999 Lawrence Lessig argued that the balance between public and private interests, and between the free flow of expressions of ideas and knowledge and state-guaranteed control and monopolies must at all times be carefully crafted, in the interest of both the society and the economy of a country, and that present tendencies favor monopolies and control too much (Lessig 1999). Subsequently, Creative Commons was founded in 2001 by Lawrence Lessig, Hal Abelson, and Eric Eldred, with the first licenses issued in 2002 (Creative Commons 2011a). After years of continuous growth, an important milestone was the migration of the Wikipedia and other Wikimedia Foundation projects to use CC BY-SA in 2009.

CC licenses have since been upheld by courts in several cases (District Court of Amsterdam 2006, Badajoz Sixth Court 2006, Tribunal de première instance de Nivelles 2009; Landgericht Berlin 2010). Using standardized licenses thus affords both licensor and licensee a certain degree of legal safety. Furthermore, many individuals and organizations world-wide are now using these licenses. This provides significant efficiency advantages in license management when creating works that are partially based on or integrate previous works (e.g., re-use of illustrations).

Creative Commons realizes that no single license is adequate for all purposes and provides a set of licenses to cover a wide range of use cases.

A special case is the “no rights reserved” or “rights-release” license, CC0 or CCZero. Not all jurisdictions have the concept of a public domain and in some jurisdictions specific rights cannot be released. CC0, as a license, describes a degree of freedom that is as close as possible to the concept of a public domain. The CC0 license is useful when the re-use of works shall be made as easy as possible. Some copyright owners release valuable works in this way, but the majority of applications are works where the eligibility for copyright is doubtful anyways (data containing occasional free-form text comments, simple graphics, icons, etc.).

**Figure:**
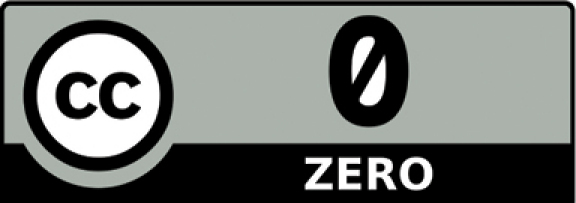
Logo of the CC Zero or CC0 Public Domain Dedication License – “No Rights Reserved” (CC BY, Creative Commons)

All other current CC licenses are combinations of four conditions, each represented by a concise summary plus either specific clauses or modifications in the full legal code.

All current CC licenses (except CC0) contain the “Attribution” condition (abbreviated “BY”), requiring appropriate attribution of the creators of a work. In the case of derived works, this condition also requires clear statements or methods to enable the user to understand the kind of changes made during a derivation. The Attribution condition is similar to the scientific community standard that all information sources must be appropriately cited. The community standard, however, refers to the attribution of ideas or data, whereas the CC BY license refers to the creative form.

**Figure:**
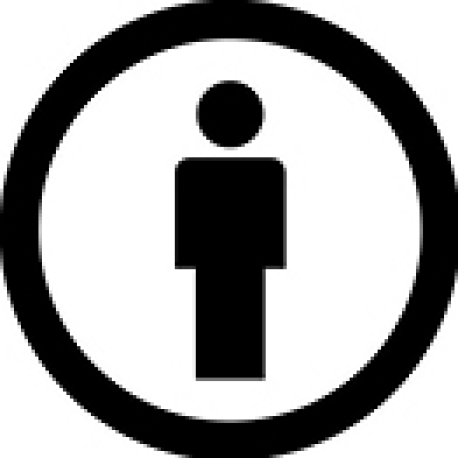
Icon of the Attribution (= BY) condition (CC BY, Creative Commons)

In biodiversity, the attribution rule is widely accepted. An exception is the case of data or metadata publishing where it is controversial whether a legally binding attribution requirement may be an obstacle to re-use. For example, the Europeana Foundation recently changed their Data Access policy for textual metadata describing multimedia objects published through the Europeana portal from something resembling CC BY-NC to CC0 (Europeana Foundation 2011). Those favoring CC0 or Open Data Licenses see problems with any legal approach to enforce norms of Attribution, Share Alike, or other terms on data or metadata (Science Commons 2011). For example, data aggregation and inheritance may lead to unmanageable attribution and licensing stacks. Furthermore, the borderline between non-copyrightable factual information and copyrightable works (e.g., sufficiently originally creative prose inside a database, or the information model as a whole) is blurred. Conversely, a license requiring attribution greatly increases the willingness of individuals and organizations to release textual data that are research results and should be properly attributed. Without it, large volumes of valuable data may remain unpublished.

The “Share Alike” condition (abbreviated “SA”) allows the distribution of derivative works, but requires that all such works must also be shared under the same conditions: “If you alter, transform, or build upon this work, you may distribute the resulting work only under the same or similar license to this one.” The effect is that licenses with the SA condition “spread” into derivative works; the biological metaphor of a “viral license” is often used here.

**Figure:**
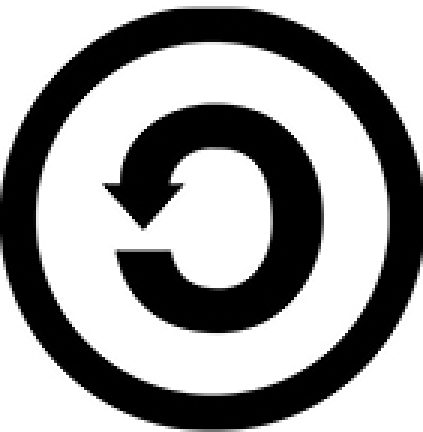
Icon of the “Share Alike” (= SA) condition (CC BY, Creative Commons)

In contrast to “Share Alike”, a creator may prevent the distribution of derivatives of a work by adding the “No Derivative Works” condition: “You may not alter, transform, or build upon this work” (abbreviated “ND”). This condition does not prevent simple format changes: It is the creative work that is protected, not a particular digital representation. The license clarifies this in “... rights may be exercised in all media and formats whether now known or hereafter devised … include the right to make such modifications as are technically necessary to exercise the rights in other media and formats, but otherwise you have no rights to make Adaptations.” This clarification allows for the creation of lossy conversions (e.g., creating smaller images as needed for small-screen media, or converting a lossless png-image into a lossy jpg-image as needed for mobile media), as long as the new representations remain truthful to the original work. In the case of tiny image thumbnails, the latter may no longer hold and the thumbnail may have to be classified an abridgment or condensation.

**Figure:**
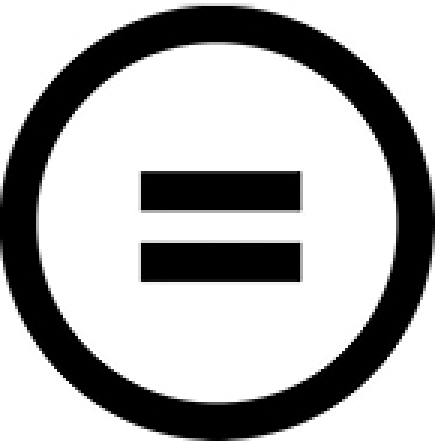
Icon of the “No Derivative Works” (= ND) condition (CC BY, Creative Commons)

The definition of “Adaptation” in the CC license is very far-reaching and the ND condition prevents many desirable uses: Cropping images or videos, adding arrows or lettering, translating into another language, creating time-synched relations between a video and other media, and (with the exception of collections of works) the use of images in a new context. Even displaying ND licensed images in lectures or presentations may be prohibited. The license explicitly names synching of audio with video as an example of prohibited use of ND licensed works and a presentation is synching one media (e.g., an ND-licensed graph from a publication) with a live audio stream. While the ND condition may have useful applications for some works of art, we recommend avoiding it for copyrightable forms of biodiversity research documentation. To describe it by a biological metaphor: the ND license is sterile and cannot spread (Katz 2006).

The final condition is the “Non-Commercial” condition (abbreviated “NC”). This condition is widely used, but also often inadequately understood. The main topic of this article is the analysis of the implications of using this condition.

**Figure:**
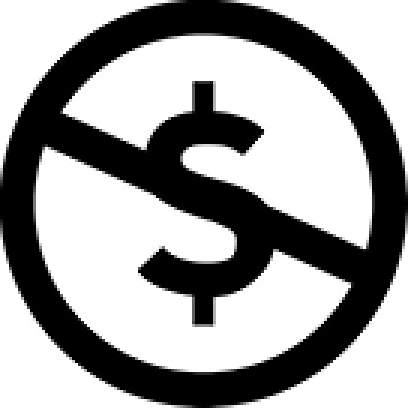
Icon of the “Non-commercial” (= NC) condition (CC BY, Creative Commons)

## The “non-commercial” condition

The short description of the non-commercial condition of Creative Commons licenses is that one “may not use this work for commercial purposes”. The full license text is:

*“You may not exercise any of the rights granted to You in Section 3 above in any manner that is primarily intended for or directed toward commercial advantage or private monetary compensation. The exchange of the Work for other copyrighted works by means of digital file-sharing or otherwise shall not be considered to be intended for or directed toward commercial advantage or private monetary compensation, provided there is no payment of any monetary compensation in connection with the exchange of copyrighted works.” (http://creativecommons.org/licenses/by-nc/3.0/legalcode)*

The full text no longer uses the term “commercial purposes”, but only the concepts of “intent or direction” and “commercial advantages”. To our knowledge, the concept of “commercial advantages” is at present neither defined by CC nor in the law of most countries (Keller and Mossink 2008; Wilson-Strydom 2009 p. 15). Between 2006 and 2008, a document “Proposed Best Practice Guidelines to Clarify the Meaning of Noncommercial” was developed on the CC wiki under “DiscussionDraftNonCommercial_Guidelines”. This was replaced in 2008 () with a link to a report "Defining Noncommercial" (Creative Commons Corporation & Netpop Research 2009). This report surveys the frequency distributions of various interpretations of the terms "commercial use" and "non-commercial use", mainly by U.S.A. Internet users. The survey confirms that significantly differing interpretations of "non-commercial" exist. The majority of users tend to identify "commercial" with "for profit". However, the study also shows that "uses by organizations, by individuals, or for charitable purposes are less commercial but not decidedly [considered as] noncommercial" (ibid., p. 73). Furthermore, the use of works surrounded by or connected with advertisements is largely considered commercial (score 82.6 to 84.6 on a scale of 0 = non-commercial and 100 = commercial). Many people will interpret it as acceptable to use a work licensed as non-commercial in combination with advertisement for cost-recovery, while others will not. A major implication from this study is that the definition given in the CC license is ambiguous, since both sides believe that the CC NC license term is "essentially the same as" or "compatible with" their definition (ibid., p. 11).

In practice, the interpretations range from considering editorial use of images in a for-profit journal as non-commercial (e.g. the interpretation by Wired magazine, see Benton 2011a) to disallowing any use where money is exchanged, regardless whether for cost recovery or not. The question as to how “non-commercial” will be interpreted in court is largely unresolved. Given the large number of potentially contentious licensing cases (e.g., Prodromou 2005; Benton 2011a), a similarly large number of court decisions in relevant jurisdictions will be required. Until this is achieved, any long-term project that considers the use of CC NC licenses will require a careful assessment of legal risks. We present here some insights we have gained in our own risk management analysis, so as to inform the decisions of others.

Formally, the word “commercial” means “referring to commerce”, which in turn may be defined as, for example: “1. the activity embracing all forms of the purchase and sale of goods and services” (Collins 2003). The term “commercial” is thus not directly linked to the concept of making profits. A non-profit enterprise that buys and sells services is a commercial enterprise according to this and many other definitions. It can consequently obtain commercial advantages, e.g., by using images for a public awareness campaign under a free license rather than paying for them on the market. Different interpretations exist: “non-commercial” may be identified with “non-profit” (summarized, e.g., by Wilson-Strydom 2009) or it may be identified with “directly making money” (Kleinman 2008, ignoring commercial advantages that only later lead to monetary profits). However, licensors that intend to apply permissive interpretations of the NC license often feel obliged to clarify their point in a license interpretation statement (e.g., Massachusetts Institute of Technology 2011; Smith 2011; or examples given in Keller and Mossink 2008).

Importantly, the NC license does not refer to the status of potential users at all; focusing solely on the manner in which a work is used. Both for-profit and non-profit organizations may use NC licenses. However, non-profit organizations probably need to rely on factors other than their status to decide whether they may use NC-licensed works.

### Monetary compensation and commercial advantages

The CC NC condition distinguishes between (1) a general definition of activities allowed under the license and (2) the special case of “the exchange of the work for other copyrighted works”. In the first case, “non-commercial” is defined in the NC condition by two elements:

a) “no private monetary compensation” (i.e., any kind of payment to the licensee by a third party) and

b) “no commercial advantage” to the licensee (i.e., any direct or indirect non-cash-profit, potentially including profits in reputation [e.g. through sponsoring] or savings of expenses [one does not have to buy a copy of the work in the shop...]).

The second case of exchanging copyrighted work does allow commercial advantages, focusing only on monetary compensation. The introduction of a special case stresses that (a) absence of “monetary compensation” is a core principle that is upheld in all cases, and (b) that any form of “commercial advantage” is a binding principle for all other activities than exchanging copyrighted works.

The authors further believe it reasonable that “compensation” includes both full and partial cost recovery.

### Primary or secondary intent

All evaluations of intent only concern the user (licensee), not the copyright owner (licensor). The latter may well have commercial motives when releasing material under an NC license (see, e.g., Benton 2011a).

With respect to the licensee, the availability of the license does not depend on the type of legal entity, but on the context and goal of the activity in which the work is reproduced or re-used. The license specifies that it excludes activities that are “primarily intended for or directed toward commercial advantage”. Deciding which “intention” or “direction” is the primary one is the main focus of controversy.

For example, a charitable non-profit organization may sell a calendar with CC-NC-licensed images as a means to raise funds. This is considered to be commercial use even by permissive interpretations of the NC-clause (e.g., Kleinman 2008), despite the fact that the ultimate intention for the funds is a charitable cause. But what about a general brochure, distributed free-of-charge? Increases in the membership base or in public recognition translate into a commercial advantage in the form of higher income through membership fees or voluntary contributions. To some extent, non-profit organizations compete with each other for donations and funds that the members of the public are willing to spend on membership fees. If a non-profit nature conservation organization uses an NC-licensed image in an advertisement brochure and the paid membership increases, it could be argued – similarly to the case of the calendar – that this use of the licensed work was primarily intended and directed toward commercial advantage.

In the case of for-profit companies, a commercial advantage can be assumed to be the primary goal in the majority of cases. Still, for example a for-profit journal, university or hospital may have a charter or mission statement that establishes charitable purposes as its ultimate goal, making the assessment of primary intent a non-trivial one.

The principle of primary intent does help with the question of cost recovery. Rutledge (2008) argues that the NC license allows for all forms of monetary compensation that relate to recovery of costs, such as printing, postage, and even salaries, since cost recovery cannot reasonably be assumed to be a primary commercial motive. While this is a reasonable position in connection with monetary cost recovery, it remains doubtful whether it also eliminates concerns about gained or lost non-monetary commercial advantages.

In general, “intent” can be problematic to assess. Legal case history for assessing the non-commercial (or non-profit) status of individual actions in which an NC-licensed work is used is probably limited to District Court of Amsterdam (2006). However, a rich case history is available in most jurisdictions for the analogous case of assessing the non-profit or charity status of organizations for taxation purposes. Similarly to the CC NC licenses, such assessment goes beyond a simple calculation of profits. A non-profit organization may make losses in one year and profits in another without threatening its non-profit status, and a for-profit organization making losses several years in a row cannot simply claim a “non-profit” status for taxation purposes. Taxation status is typically assessed by a complex set of rules, governed by law, but in detail often defined by individual taxation authorities. Despite a long case history and detailed assessment rules, it is possible that an organization achieves non-profit status in one taxation district, and fails to do so in another. Assessing the non-commercial intent of individual actions in court may be vastly more complicated.

## Re-use options of NC-licensed works

The CC NC clause defines wide-ranging limitations to protect the commercial interests of the creator or copyright owner of a work. In our understanding, the following conditions determine whether an NC-licensed work may or may not be re-used:

1. Any natural or legal person or organization, including commercial enterprises, may exercise licensed rights over an NC-licensed work. The ability to re-use, copy, or derive from a work depends on the context and goal of the activity, not on the type of legal entity exercising the rights.

2. Charging money for the work as a means to obtain a profit is clearly prohibited; there will be little doubt that this has been the primary intent when exercising the rights granted by an NC-license.

3. Charging money for the work as a means to recover cost seems initially prohibited. The license text uses the term “compensation” rather than “profit” or “gain” and stresses that this principle is to be upheld even in the case of exchanging works. However, cost recovery is likely permitted if a different primary intent and direction can be demonstrated.

4. Regardless of profit or cost recovery, the use of a licensed work may lead to (non-monetary) commercial advantages. Arguably, most uncharged uses of a work can be interpreted as an advertisement, and increased public recognition is generally seen as an advantage for any legal entity participating in commerce. Users of NC-licensed works must thus demonstrate that the use is neither primarily intended for, nor directed towards such increased recognition.

5. One might perhaps be in doubt whether the concept of “commercial advantages” might be applicable to private individuals as well: For someone working as a gardener or professional biologist, the action of re-publishing a biodiversity-related NC-licensed work could be assumed to be directed towards financial advantages (e.g., self-advertisement to improve the chances of finding new employment). However, the mentioning of “private monetary compensation” may be interpreted to implicitly clarify that the (broader) concept of “commercial advantages” is not to be applied to private individuals.

6. In most cases, the allowed use of an NC-licensed work therefore hinges on the question of whether the advertisement effects are primary or not. The following thought examples may demonstrate that the legitimacy of using NC-licensed content may be difficult to decide. Assume that an NC-licensed image is used in these contexts:

a) A large for-profit soft-drink producer runs an advertisement campaign “better drinks for a more joyful life”.

b) A large for-profit company advertises their products with “50 cents from each purchase buys and preserves a piece of Amazonian rain forest”.

c) A large non-profit nature conservation organization runs an advertisement campaign to increase its paying membership base, with the ultimate goal to increase its financial and political abilities to serve the cause of nature protection. However, by doing so, it is competing with other nature conservation organizations.

Most readers would probably consider cases a) and b) a license violation, but formally all organizations might claim that this particular action is primarily intended for and directed toward a public benefit. Thus, with different degree of likelihood, in each of these cases, a court might or might not decide that the advertisement is directed towards commercial advantages, making the use of the work a violation of the license terms.

## Software

Software programs are copyrighted works and can in principle be released under CC licenses. This is, however, not recommended (Creative Commons 2011b). Unlike most other copyrighted works, software can be used as a tool to create other works. With respect to NC licenses, the condition “You may not exercise any of the rights granted to You in Section 3 above in any manner that …” implies that software licensed under such a license (e.g., xper2, Ung et al. 2010; FRIDA, Martellos et al. 2010; or OpenKeyEditor, van Spronsen et al. 2010) may not be used to produce creative works or non-copyrightable data sets for commercial purposes.

This is not dependent on the presence of a Share Alike condition. A work created with the help of a software application is normally an independent creation. The cases where software generates derivative works are fairly limited, e.g., where software-created works are primarily derivatives of copyright materials embedded in the software (i.e. materials other than software algorithms or source code) or where the arrangement and formatting applied by the software to non-copyrightable data is actually the primary copyrightable creative component. This may indeed occur in biodiversity where data are formatted as software-generated “species pages”.

This is, however, not the primary concern with NC-licensed software. The creator of such work created using NC-licensed software may have full ownership and copyright to it, but is limited by the contractual obligations which arise from using the NC license. The critical question is perhaps: Which level of diligence in preventing commercial use of such works or data sets is required? Is it sufficient that no commercial use was *intended* at the time of creation (but may the work later be sold)? May the author give it as a present to a third party, which may then put it to commercial use? Or is the author required to prevent this from happening for all times, including binding future copyright heirs?

Following the recommendations of Creative Commons, we advise that the only Creative Commons license suitable for software is the CC0 rights release license. Dedicated software licenses should be used in all other cases.

## License compatibility

Works licensed under CC licenses that do not include the NC condition are naturally available for non-commercial use. However, a common misconception is that such works and those licensed with an NC condition can always be mixed in a derivative work, creating a new work under the more restrictive license.

While it is possible, e.g., to combine works licensed under CC BY-NC with works licensed under CC BY content, it is not possible to do so with works under licenses containing the Share Alike condition (e.g., the CC BY-SA license on Wikipedia text and most images). Share Alike prevents the use of a work under a more restrictive license – specifically in this case under an NC license. A derived work that combines NC-SA and other licenses must be shared under an NC license. This would be incompatible with the Share Alike license terms for an included CC BY-SA work (Katz 2006). License compatibility can be checked, e.g., with the Creative Commons Licenses Compatibility Wizard (Creative Commons Taiwan 2011).

The problem of license incompatibility may also arise when licensors, recognizing the problems with the CC NC license, amend it with their own definitions (see, e.g., Massachusetts Institute of Technology 2011, Smith 2011, or examples given in Keller and Mossink 2008). In the case of the CC BY-NC-SA license, if two licensors annotate a license in contradictory ways, these two licenses may be incompatible with each other (while each may remain compatible with unmodified and unspecified CC BY-NC-SA licenses).

License incompatibility problems also surface in relation to license models outside Creative Commons. Only the CC BY and CC BY-SA licenses (but not CC BY-ND, CC BY-NC, or CC BY-NC-SA) meet the criteria of openness that are used to determine compatibility with, e.g., software licenses laid out in each of:

• Open Knowledge Definition (Open Knowledge Foundation 2006),

• OSI Open Source Definition (Open Source Initiative 2004),

• Definition of Free Cultural Works (Möller 2008; Möller and Anonymous 2007ff),

• the GNU Free Software Definition (Free Software Foundation 2010),

One option to avoid such license incompatibility is to remove the “Share Alike” clause, insisting on attribution alone (CC BY, e.g., Benenson 2008). This further increases the dissemination and reusability of a work. However, this also allows the possibility that derived works may not be “given back”, i.e. that works derived from free and open content may not themselves be open.

In light of the incompatibility between the most frequently used CC licenses (CC BY-SA and CC BY-NC-SA), a highly relevant question for biodiversity information dissemination is: Which combinations of works under different licenses result in a “collection” (in which cases the above CC licenses may be mixed) and which create an illicit derivative work or adaptation? In our experience, the (unambiguously incompatible) case of combining two texts seamlessly into a new work, such that the borders between the original works can no longer be traced, is not very relevant for the biodiversity domain. Typically, original works remain delimited and authorship and license of the parts documented. A web page with a gallery of images where the license and creators of each image is annotated will certainly be a collection. The same should apply for similarly clearly separated blocks of text, or combinations of text and image blocks.

Further, copyright law does not refer to digital representations but to abstract works. Thus, whether an image gallery is composed of separate files bound together by a web page, or whether the elements have been combined into a single file (e.g., because of the need for non-rectangular cropping or connecting elements) should not change the status as an “image collection”, provided the parts remain individually recognizable and attribution and license individually documented.

However, the ways in which media (sound, images, or video) or text are combined in many biodiversity projects go significantly beyond image galleries or the traditional collection examples (“encyclopedias and anthologies”) mentioned in the Creative Commons license text. Images and other media are often closely embedded and integrated with corresponding text. The CC licenses do anticipate creative arrangements. Collections may “by reason of the selection and arrangement of their contents, constitute intellectual creations” (http://creativecommons.org/licenses/by-sa/3.0/legalcode). Within biodiversity, the Encyclopedia of Life (EoL, http://eol.org) uses complex combinations of CC BY-NC-SA and CC BY-SA material. However, EoL has license agreements with its contributors allowing for use on EoL independent of the Creative Commons licenses. A more relevant example may thus be the complex ways in which Wikipedia occasionally combines text under CC BY-SA with images under various open content licenses share-alike-licenses, e.g., some images being licensed exclusively under the GNU Free Documentation License.

## Licensing patterns

The majority of large-scale global collaborative projects promote the use of “free” or “open content” licenses. Free and open are often used interchangeably, but we will use them here in the sense that free just means that accessing the information does not involve costs beyond those of accessing the web, whereas open shall refer to the absence of “non-commercial” and “no-derivative” conditions.

The distribution of the various CC licenses depends on the cultural and commercial context of the various communities. Statistics maintained by Creative Commons to record various license uses show that 60% of all CC-licensed works in 2010 (primarily from Flickr and Yahoo, Linksvayer 2011a) are under non-free CC licenses (with ND, NC condition). The proportion of open licenses is slowly increasing over the years, however (Linksvayer 2011a, b).

Within the context of biodiversity, the proportions of non-open licenses are similar. A quality control web service (Morris 2009) showed that 76% of nearly 95 000 CC-licensed images in the Flickr EoL Images Group (Flickr 2011) had NC licenses on them. However, the average for EoL may be different, since EoL had other media sources in addition to Flickr. For the Atlas of Living Australia (ALA), 34 out of 58 CC licensed data sets include a non-commercial term (58,6%; 28 CC BY-NC, 6 CC BY-NC-SA, pers. comm. Miles Nicholls).

By contrast, a Google search reveals that among the PubMed Central corpus (U.S. National Library of Medicine 2011a), the open content CC BY license was chosen nearly three times as often as all NC licenses combined (Mietchen 2011).

The “Defining Noncommercial” report (Creative Commons Corporation & Netpop Research 2009) shows that the vast majority of copyright holders publishing works under a non-commercial license are willing to interpret the license in a liberal sense, e.g., accepting the use in combination with cost compensation or as advertisement by educational or non-profit organizations (see also Dobusch 2011). However, organizations planning to re-use NC-licensed works are a) forced to accept a legal litigation risk and b) are restricted due to license compatibility issues in the case of licenses containing the Share Alike condition. As a result, many public education projects like Wikipedia, OpenStreetMap, Wikibooks, Wikiversity, Connexions, Encyclopedia of Earth Citizendium, WikiEducator, Appropedia, etc. have decided that NC licenses are not suitable for them. Non-open licenses like CC BY-NC-SA seem to dominate in terms of number of published items, whereas open content licenses (CC BY, CC BY-SA) may dominate in terms of re-use.

By their very nature, the severe constraints on NC-licensed works reduce the societal benefits arising from those works (Möller and Anonymous 2007ff). Non-commercial licenses do not create the same kind of synergistic, agile, collaborative environment or re-use and continuous improvement that open content licenses create.

At the Creative Commons Global Summit 2011, CC representative Mike Linksvayer stated (Linksvayer 2011b; Dobusch 2011): “… the NC condition still sounds very appealing to many creators and is thus probably overused by those without existing revenue streams to a project. This could in turn lead to an under-use of non-NC licenses, which realize far more value since there are projects (e.g., Wikipedia) that rely on free licenses to exist.”

The EU project ViBRANT (Virtual Biodiversity Research and Access Network for Taxonomy) is based on a combination of multiple platforms (Berendsohn et al. 2011). In its first years it recommended the use of a CC BY-NC-SA license on its Scratchpads web publication platform (Smith et al. 2009; Smith et al. this volume), the CC BY-SA license on the MediaWiki (biowikifarm, Hagedorn et al. 2010) platform, and CC BY (with major contributors choosing CC BY-NC-SA, however) on the CDM platform (Berendsohn 2010). The present paper is partly motivated by observing the resultant incompatibilities. For the future, contributors employing the ViBRANT Scratchpad 2 platform to be deployed in 2012 will be encouraged to use an open license. A CC BY license will be the default for new content, although users may choose other licenses, including those with a non-commercial clause.

## Summary and conclusions

Creative Commons licenses are not antagonistic to copyright – they are based on it. A violation of a CC license is a copyright violation. CC licenses replace individual contracts (that the copyright owner and the user of a work may negotiate) with a standardized license. Managing individual licenses incurs a high legal and management overhead (which induces many publishers not to negotiate licenses, but rather to demand total transfer of copyright). The availability of a set of such standard contracts for a spectrum of use cases is an important feature of CC licenses.

The Creative Commons Non-Commercial (CC NC) licenses exclude re-use scenarios leading to monetary profits or other commercial advantages (increased notability, etc.). It thus effectively protects copyright owners whose income depends on commercially licensing their works. NC licenses therefore are an important instrument to contribute a work to causes in which a third party’s gain does not diminish the revenue of the copyright holder. Contributing marketable works under an NC license is a laudable act.

Nevertheless, the NC licenses are also deceptive. The phrases “creative commons” and “non-commercial”, together with the strong tendency in colloquial language to (incorrectly) identify “commercial” with “profit” and “non-commercial” with “non-profit”, may suggest that releasing works under this license contributes to a “non-commercial commons” that is easily re-usable for all non-profit-minded entities. This, however, is not the case. NC licenses come at a high societal cost: they provide a broad protection for the copyright owner, but strongly limit the potential for re-use, collaboration and sharing in ways unexpected by many users:

1. While some interpretations plausibly argue that in public perception non-commercial and non-profit are widely seen as closely related, a public misconception is likely to be irrelevant in a court case. Most non-profit organizations or charities engage in commercial activities like buying and selling goods and services. They are potential buyers of copyrighted works; allowing them to re-use a work free of cost potentially diminishes the commercial revenues of the copyright owner. Copyright owners licensing their works under an NC license might well intend to apply a strict interpretation of non-commercial, so as to not lose potential profits from this market sector.

2. The phrase “commercial advantages” covers a very broad spectrum of activities, including advertisements, sponsoring, fund-raising, or any other activity that improves brand recognition or public relations of an organization or individual. The fact that this is widely ignored (Wild 2011; Benton 2011b) does not make it legal.

3. The CC Attribution-NC-Share-Alike license is incompatible with the CC Attribution-Share-Alike license. NC licenses therefore cannot be used on major collaboration platforms like Wikipedia or Wikimedia Commons (Möller 2007; Wikimedia Commons 2009).

4. The decision whether an activity is “primary” or “secondary” will be difficult to argue and decide in courts. For example, fundraising will primarily be directed towards monetary gain. This, however, may ultimately be intended to hire a person to work in nature conservation. Risk management will require careful documentation of intent and actions while running a project involving the re-use of NC licenses.

In conclusion, the licensing concepts “commercial advantages”, “primarily”, and “intent” are difficult to define and assess, resulting in a significant risk of litigation to private persons as well as organizations that use works supplied under an NC license. Being an educational or non-profit organization does reduce the likelihood of litigation in terms of frequency (because many licensors accept such use). For a given litigation, however, we fear that a substantial risk exists of losing the case.

Individual claims of license violation brought forward by copyright owners can often be settled out of court. In some countries an internet platform may further be covered by some form of a copyright infringement liability limitation privilege (e.g., requiring a take-down-notice). However, another threat to project sustainability may come from competitors in the publishing business which may consider a particular use of NC licensed works illicit. Depending on the specifications of unfair competition laws in a given country, they may attempt to acquire an injunction stopping any “license violations” that lead to unfair competitive advantages. Should they succeed, this would then require to remove all NC-licensed materials from a project.

In addition to managing legal risk, projects considering to re-use, disseminate, or create derived works under NC licenses may also need to evaluate their future project development options. For example, collaboration needs and cost-compensation schemes for the provision of content on an Internet platform may differ from needs and schemes for the provision of works in print, on offline media like CDs or as smartphone applications. Creative Commons recommends seeking individual permissions for any use of NC licensed content that may be controversial as to whether it is commercial or not (Linksvayer 2009). Especially if content is created and re-used collaboratively on a platform that leads to tight integration of the contributions, it may not be practical to later reverse the choice of license: All contributors would have to be contacted for a new negotiation. In our experience, the proportion of contributions which cannot be reached, have lost interest, never meant to market their contribution (having misunderstood the purpose of NC), or are unwilling to consent is relatively high. The contributions of these, which may be intermediate revisions of a text, then have to be laboriously removed.

Creative Commons is aware of the problems with NC licenses. Within the context of the upcoming version 4.0 of Creative Commons licenses (Peters 2011), it considers various options of reform (Linksvayer 2011b; Dobusch 2011):

• hiding the NC option from the license chooser in the future, thus formally retiring the NC condition

• dropping the BY-NC-SA and BY-NC-ND variant, leaving BY-NC the only non-commercial option

• rebranding NC licenses as something other than CC; perhaps moving to a “non-creativecommons.org” domain as a bold statement

• clarifying the definition of NC

The authors of this article view NC licenses as a valid choice. Without them, many works would not be publicly licensed at all. However, NC licenses should no longer be presented as an obvious or easy choice. Rather than abandoning NC licenses, we would prefer Creative Commons to rename and rebrand them, reducing the mismatch between the actual consequences and the expectations generated by terms like “non-commercial” and “creative commons”. A combination of: 1) a name like “Non-Open Commons: Attribution-Commercial Rights Reserved, NCC BY-CR”, 2) explanations on the license chooser highlighting potential misunderstanding, and 3) a visual design change in the license display of the short and long license texts (e.g. red-gray striped instead of yellow) might better communicate the actual consequences. Independently, a clarification of the license terms, stating that uses of NC-licensed works by organizations certified as charities or non-profit organizations for the purpose of taxation in their country of residence are always appropriate, might help to reduce the risk of using NC-licensed works. Such a clarification should not change the NC license by making the use of NC licensed words dependent on the status of the user. It should only clarify that certification by taxation authorities is a sufficient test to evaluate primary versus secondary intentions. Finally, a license update should attempt to clarify the borders of collections, and contain guidelines how to document the license status of collections containing a mixture of incompatible licenses.

Given such changes, we hope that the preference for NC-licensing by publicly funded organization who can afford to provide materials into an Open Content Commons is waning. We believe NC licenses should not be used for the dissemination of results from publicly-funded organizations or research projects. The public rightfully expects a return for its investments in the form of re-usable digital content. This is the new digital infrastructure of the information era.

With respect to individual users, the major providers of collaborative biodiversity platforms could immediately start to make the choice of NC licenses less deceptive. A choice of licensing options should be given and the NC license should be present in ways that avoid raising false assumptions. “All Commercial Rights Reserved, most use by for-profit as well as non-profit organizations prohibited” is a better representation of the effect of the license.

Open content licenses such as CC BY (used by many Open Access publishers) or CC BY-SA (used, e.g., by Wikipedia) will enable a much wider re-use of a contribution and increase the efficiency of non-profit organizations in informing and educating others about biodiversity and nature conservation. We therefore recommend copyright owners to balance the negative impact of the non-commercial restriction on open knowledge dissemination, collaboration and ease of re-use against income which may be lost. In many cases, the potential profits from commercial use are comparatively low or irrelevant.

However, a publisher may indeed, with appropriate citation of the authorship, use an openly-licensed work in a book that generates a profit. The resulting dissemination of knowledge on biodiversity, regardless of profits, may well be in the interest of biodiversity education and society in general. Open licenses like CC0, CC BY, or CC BY-SA allow the commercial and private sector to collaborate and to develop businesses based on and contributing to the digital commons (Keller and Mossink 2008, Fletcher 2011). Furthermore, open licenses will help small companies or local non-profit initiatives more than big companies. Large companies can afford to buy works and can bear high management overhead, the cost of legal advice, or the risk of litigation much better than small organizations and initiatives.

Each creator of a work considering licensing options is therefore encouraged to balance the potentially lost income against the increased benefit to society. Within our own field of biodiversity, we hope that more organizations and publishers encourage their contributors to avoid NC licenses. The “commons” of CC NC licenses is available to a few, but not to the many.
